# Supplementary material for: An atypical Arp2/3 complex is required for Plasmodium DNA segregation and malaria transmission
Source: Nat Microbiol. 2025 Jun 13;10(7):1775–90. doi: 10.1038/s41564-025-02023-6 (PMC12222016; doi:10.1038/s41564-025-02023-6)
Supplement: Supplementary file 1 — Supplementary Figs. 1–6, Supplementary Table 1 and Source data for uncropped gel images. [file 41564_2025_2023_MOESM1_ESM.pdf]

nature microbiology

Supplementary information https://doi.org/10.1038/s41564-025-02023-6

An atypical Arp2/3 complex is required for *Plasmodium* DNA segregation and malaria transmission

In the format provided by the authors and unedited

Supplementary Figures

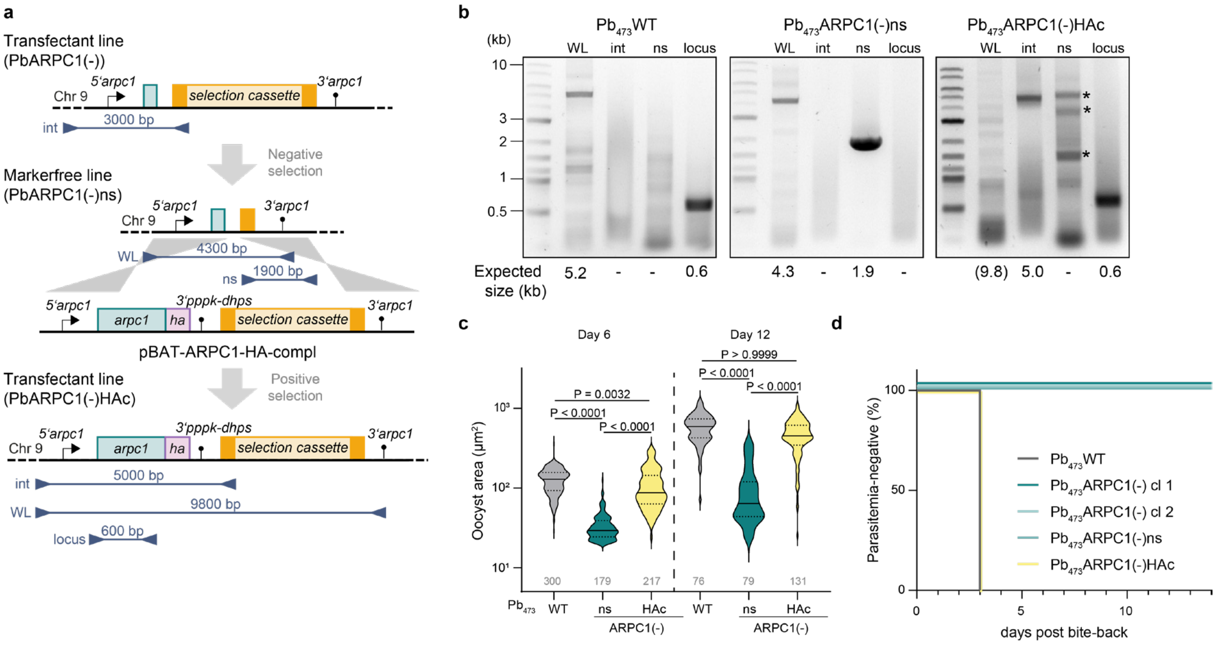

Supplementary Fig. 1: Complementation of Pb473ARPC1(-) rescues oocyst growth defect and transmission block. a, Scheme of genetic strategy. Primers (triangles) and expected amplicon sizes used for genotyping (see **b**) are indicated. Not drawn to scale. **b**, Genotyping PCR of Pb473ARPC1(-)HAc. Binding sites of the respective primers are indicated in **a** and in Extended Data Fig. 3b. The expected size of the product is indicated below the gel images. Note the presence of some unspecific amplification products in some PCRs (indicated with asterix). **c**, Oocyst area at day 6 and 12 after mosquito infection. Pooled data from 2 independent cages. Grey number above x axis indicates total number of cells/midguts analysed. Statistics: Kruskal-Wallis Test, with Dunn's post test. **d**, Parasite prevalence in mice after by-bite infection with 5-10 infected mosquitoes each. 3 mice per group.

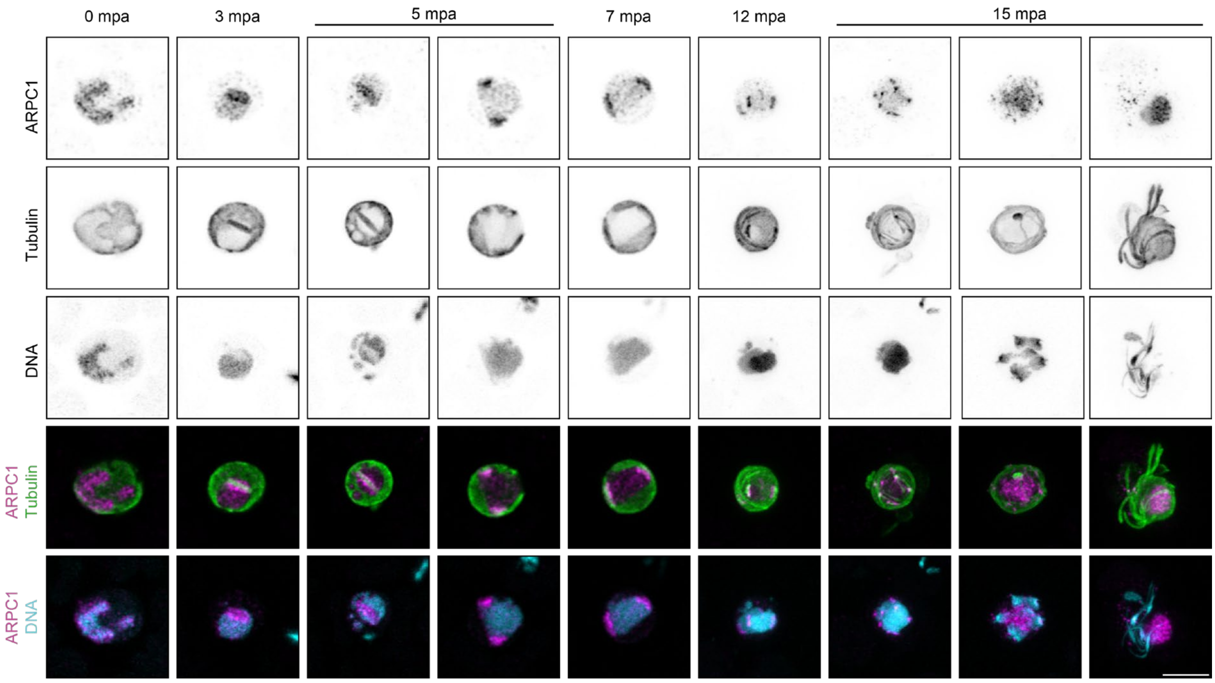

Supplementary Fig. 2: Localisation of ARPC1-GFP during male gametogenesis. Activated PbARPC1-GFP male gametocytes fixed at various time points after activation and stained for ARPC1, tubulin and DNA (Hoechst). Shown are extended panels to Fig. 2b. Representative Airyscan images of at least 5 images taken per time point. 0-7 mpa: Single slice. 12-15 mpa: Maximum Z projection. Scale bar, 5 µm.

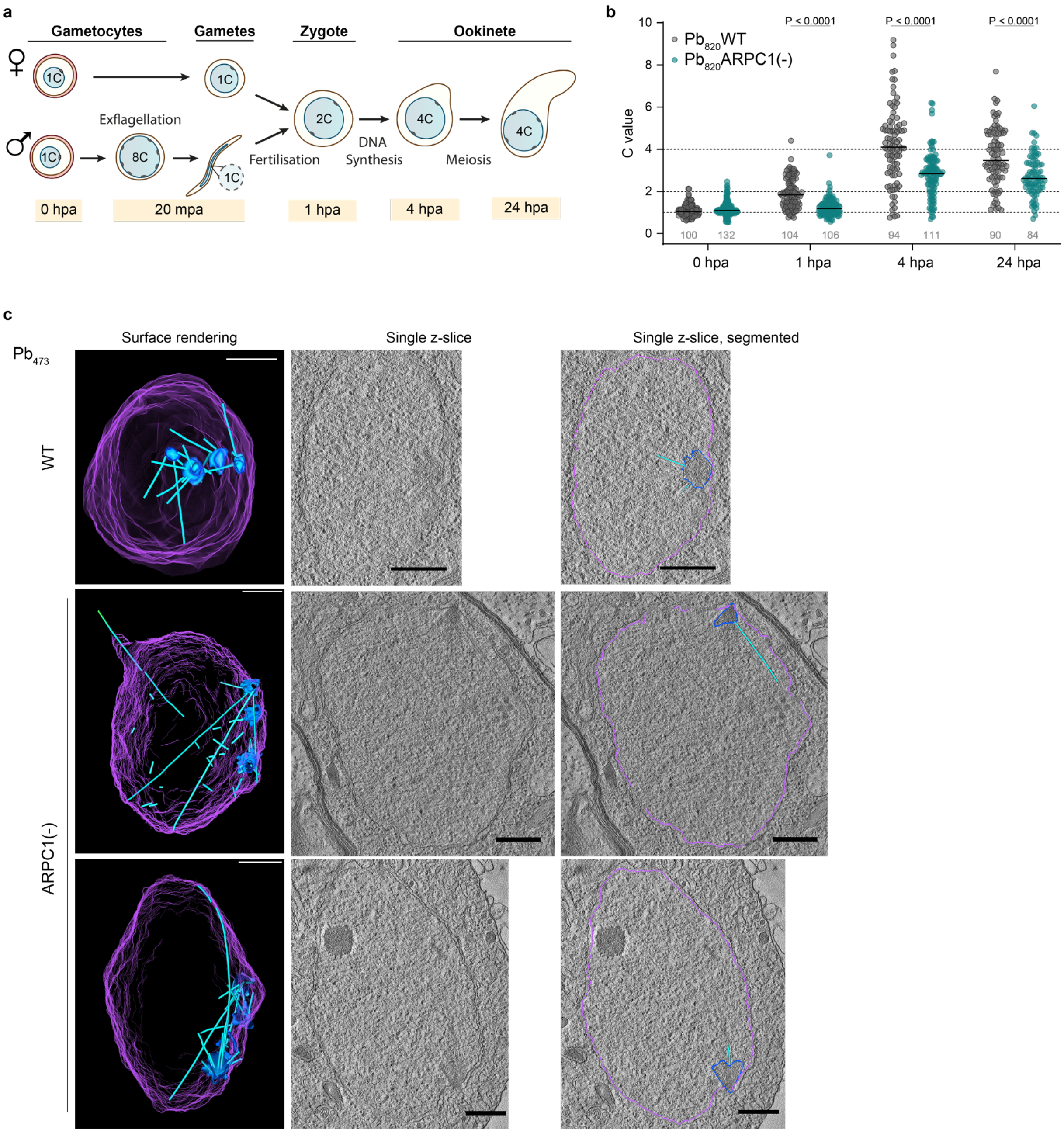

Supplementary Fig. 3: Ookinete phenotype of PbARCPC1(-). **a**, Scheme of *Plasmodium* sexual replication. C, C value (number of haploid genomes); mpa/hpa minutes/hours post activation. **b**, DNA content of female gametocytes, zygotes and ookinetes, normalised to the mean DNA fluorescence at 0 hpi. Dashed lines, expected C values at 0 hpi (C=1), 1 hpi (C=2) and 24 hpi (C=4). hpi, hours post induction. Statistics: Two-Way ANOVA, Šidák's post test. **c**, Surface-rendered 3D segmentation and example single z-slices of Pb_{473}WT and Pb_{473}ARPC1(-) ookinete nucleus tomogram. Purple, nuclear membrane; blue, MTOC; light blue, microtubules. Scale bars, 500 nm (surface rendering) and 400 nm (single sections). Both WT and ARPC1(-) ookinetes do undergo meiosis as indicated by centrosome duplication and spindle formation.

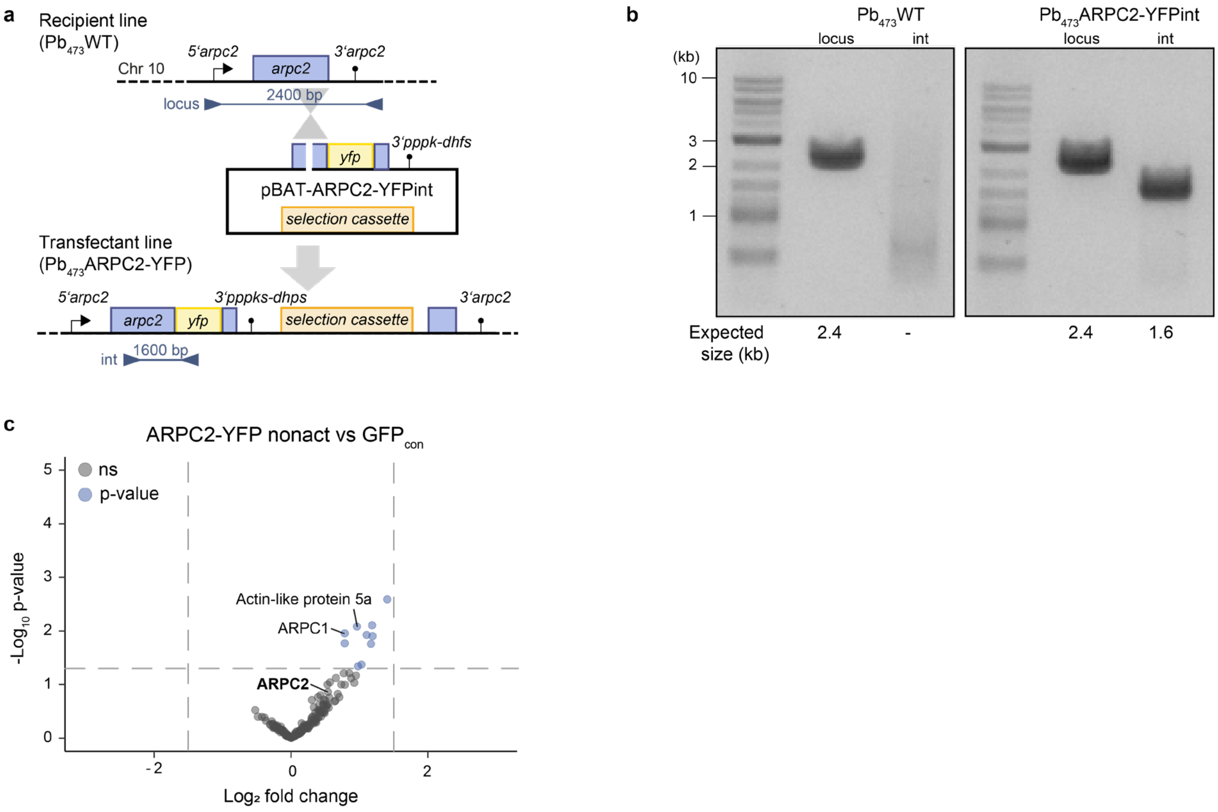

Supplementary Fig. 4: Generation of ARPC2-YFP and identification of interaction partners. a, Scheme of genetic strategy to generate Pb_{473}ARPC2-YFPint. Primers (triangles) and expected amplicon sizes used for genotyping (see **b**) are indicated. Not drawn to scale. **b**, Genotyping PCR. The binding sites of the respective primers are indicated in **a**. The expected size of the product is indicated below the gel images. **c**, Enriched proteins after pulldown of ARPC1-GFP from non-activated gametocytes in comparison to GFP_{con} gametocytes. While no hit above the fold change threshold was detected, Alp5a and ARPC1 were found above the threshold for significance.

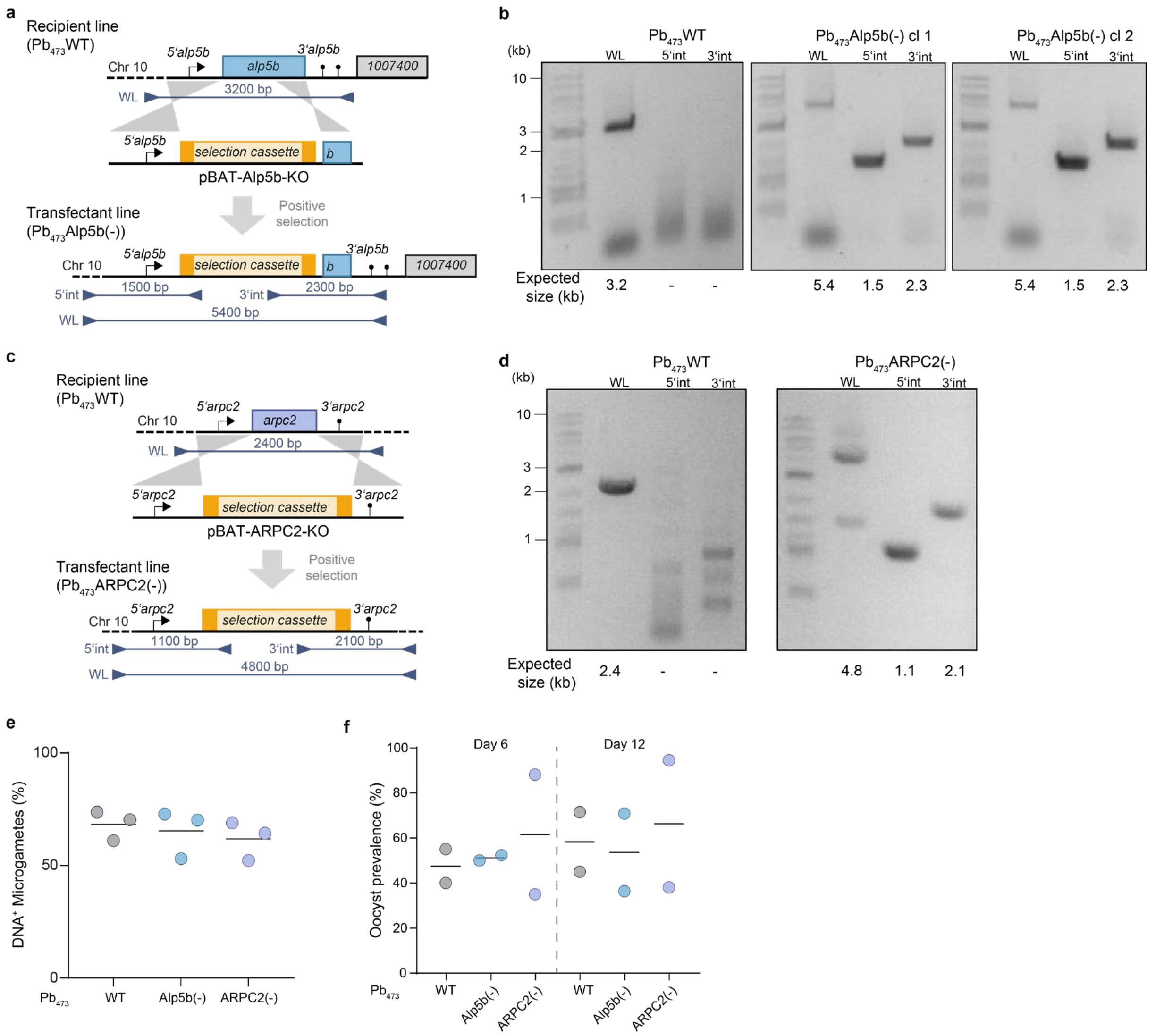

Supplementary Fig. 5: Characterisation of further Arp2/3 subunits. a,**c**, Schemes of genetic strategies to generate (**a**) Pb_{473}Alp5b(-) and (**c**) Pb_{473}ARPC2(-). Primers (triangles) and expected amplicon sizes used for genotyping (see **b**,**d**) are indicated. Not drawn to scale. **b**,**d**, Genotyping PCR of (**b**) Pb_{473}Alp5b(-) and (**d**) Pb_{473}ARPC2(-). Primers are indicated in **a** and **c**, respectively and expected amplicon sizes are shown below gels. **e**, Relative abundance of microgametes with detectable DNA signal. Each data point represents one independent experiment. **f**, Mosquito infection rate as proportion of midguts carrying oocysts. Each data point corresponds to an independent cage feed. **e**,**f**, Line indicates mean. This data phenocopies the one obtained for ARPC1(-).

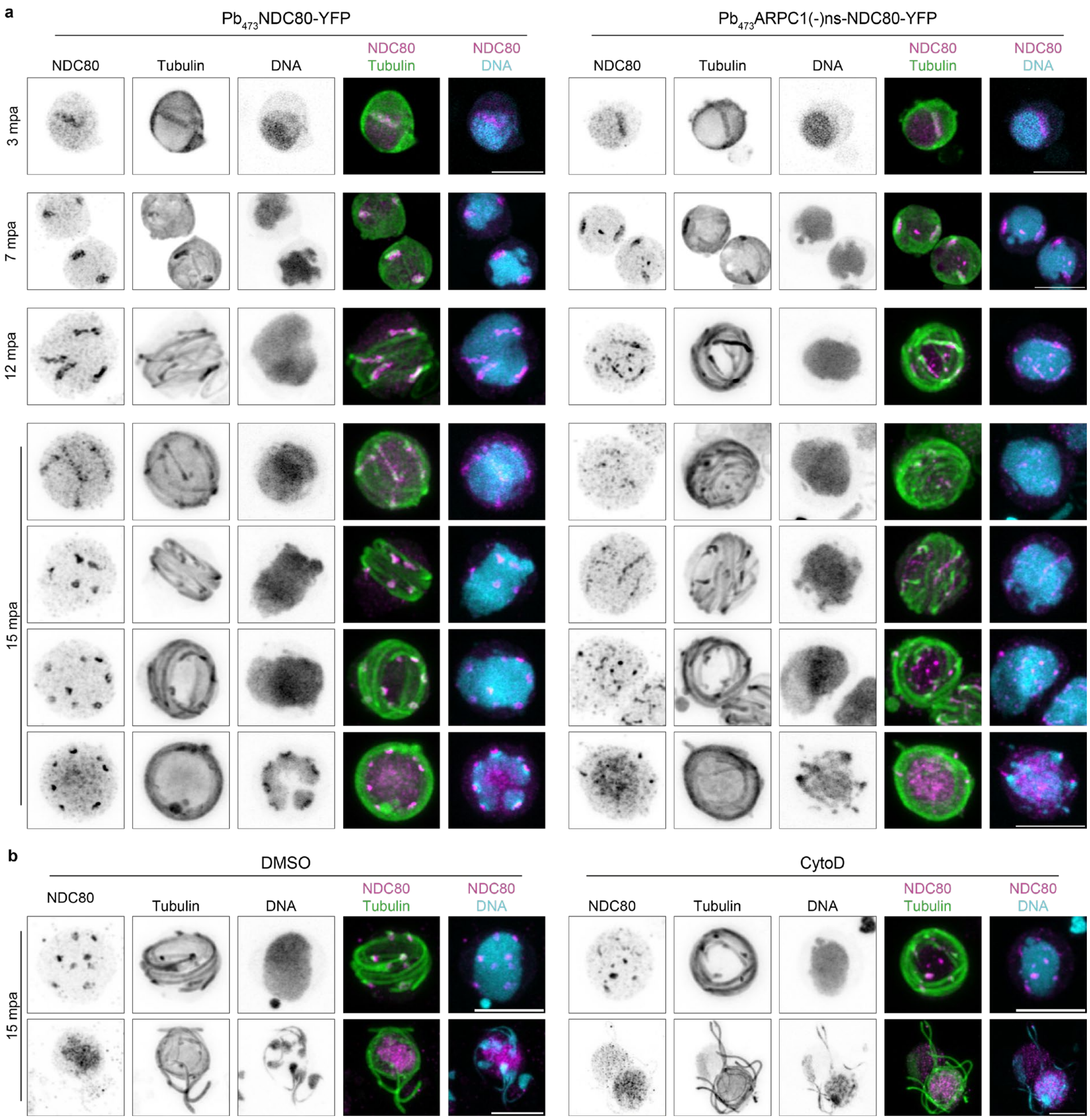

Supplementary Figure 6: Localisation of NDC80-YFP during male gametogenesis. a, Activated Pb_{473}NDC80-YFP and Pb_{473}ARPC1(-)ns-NDC80-YFP male gametocytes fixed at 3, 7, 12 or 15 mpa. Shown are extended panels to Fig. 5a and Extended data Fig. 15. 3 mpa, single slice. 7-15 mpa, maximum Z projection. **b** Pb_{473}NDC80-YFP male gametocytes activated in presence of solvent (DMSO) or 1 µM CytoD and fixed at 15 mpa. Shown are extended panels to Fig. 5d. Maximum Z projection. **a,b,** Cells are stained for NDC80-YFP, tubulin and DNA (Hoechst). Representative Airyscan images of at least 5 images taken per time point. Scale bars, 5 µm.

Supplementary Tables:

Supplementary Table 1: Summary of sporozoite numbers and by-bite infections.

Parasite line	Oocyst prevalence	Midgut spz #spz/mosquito (#mosquitoes)	Salivary gland spz #spz/mosquito (#mosquitoes)	By-bite infections positive/total mice (mouse strain)
**Pb_{820}WT**	n.d.	n.d.	16 000 (20)	3/3 (TO)
**Pb_{820}ARPC1(-) cl1**	n.d.	n.d.	0 (20)	0/3 (TO)
**Pb_{820}ARPC1(-) cl2**	n.d.	n.d.	0 (20)	0/3 (TO)
**Pb_{473}WT**	100 %	n.d.	5 700 (10)	2/2 (TO)
**Pb_{473}ARPC1(-) cl2**	67 %	n.d.	0 (10)	0/2 (TO)
**Pb_{473}WT**	83 %	n.d.	10 000 (10)	n.d.
**Pb_{473}WT**	75 %	n.d.	18 000 (10)	n.d.
**Pb_{473}ARPC1(-) cl2**	90 %	n.d.	0 (10)	n.d.
**Pb_{473}WT**	47 %	17 000 (12)	15 000 (30)	3/3 (C57Bl/6)
**Pb_{473}ARPC1(-) cl1**	75 %	0 (16)	0 (29)	0/3 (C57Bl/6)
**Pb_{473}ARPC1(-) cl2**	67 %	0 (12)	0 (30)	0/3 (C57Bl/6)
**Pb_{473}WT**	64 %	36 000 (14)	30 000 (15)	n.d.
**Pb_{473}ARPC1(-) cl1**	77 %	0 (14)	0 (20)	n.d.
**Pb_{473}ARPC1(-) cl2**	55 %	0 (20)	0 (15)	n.d.
**Pb_{473}WT**	93 %	n.d.	38 000 (15)	n.d.
**Pb_{473}ARPC1(-)ns**	83 %	n.d.	0 (24)	0/3 (C57Bl/6)
**Pb_{473}ARPC1(-)HAc**	63 %	n.d.	27 000 (16)	3/3 (C57Bl/6)
**Pb_{473}WT**	38 %	n.d.	19 000 (16)	n.d.
**Pb_{473}ARPC1(-)ns**	26 %	n.d.	0	n.d.
**Pb_{473}ARPC1(-)HAc**	42 %	n.d.	10 000 (20)	n.d.
**Pb_{473}WT**	58 %	113 500 (15)	15 000 (13)	n.d.
**Pb_{473}Alp5b(-)**	62 %	0 (15)	<100 (15)	n.d.
**Pb_{473}ARPC2(-)**	93 %	<100 (15)	<100 (15)	n.d.
**Pb_{473}WT**	43 %	56 600 (15)	14 700 (15)	n.d.
**Pb_{473}Alp5b(-)**	40 %	<100 (15)	<100 (15)	n.d.
**Pb_{473}ARPC2(-)**	37 %	140 (15)	<100 (15)	n.d.

Supplementary Table 2. (separate file)
Protein abundances and quantification after pulldown of ARPC1-GFP.

Supplementary Table 3. (separate file)
Protein abundances and quantification after pulldown of ARPC2-YFP.

Supplementary Table 4. (separate file)
Protein abundances and quantification after pulldown of AKiT7-YFP.

Supplementary Table 5. (separate file)
Primers used in this study

Supplementary Table 6. (separate file)
Source data to Supplementary Figures.

Source data – Uncropped gel images

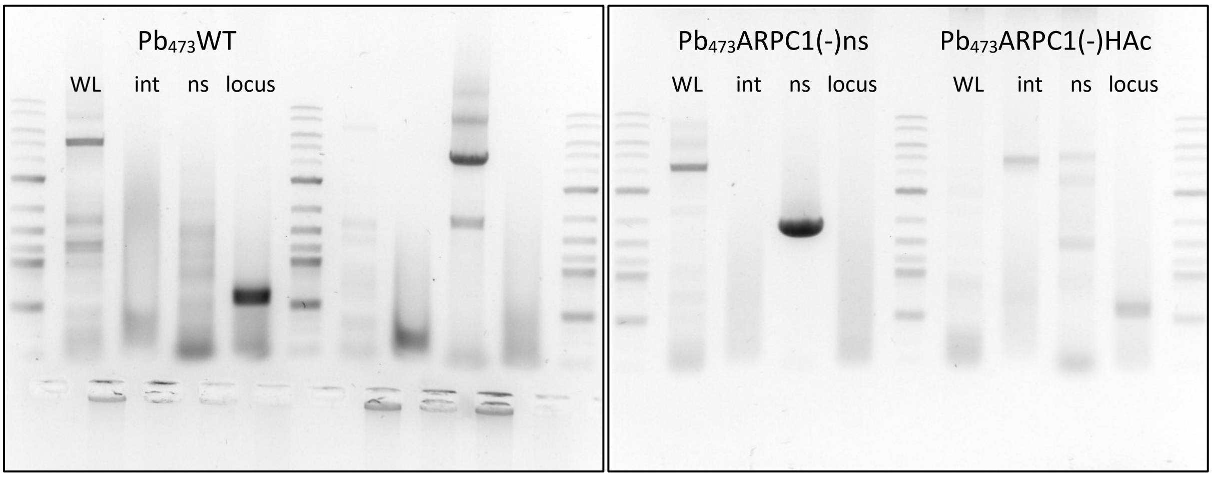

Supplementary Figure 1b - Uncropped gel image. Unlabeled lanes correspond to an unrelated project.

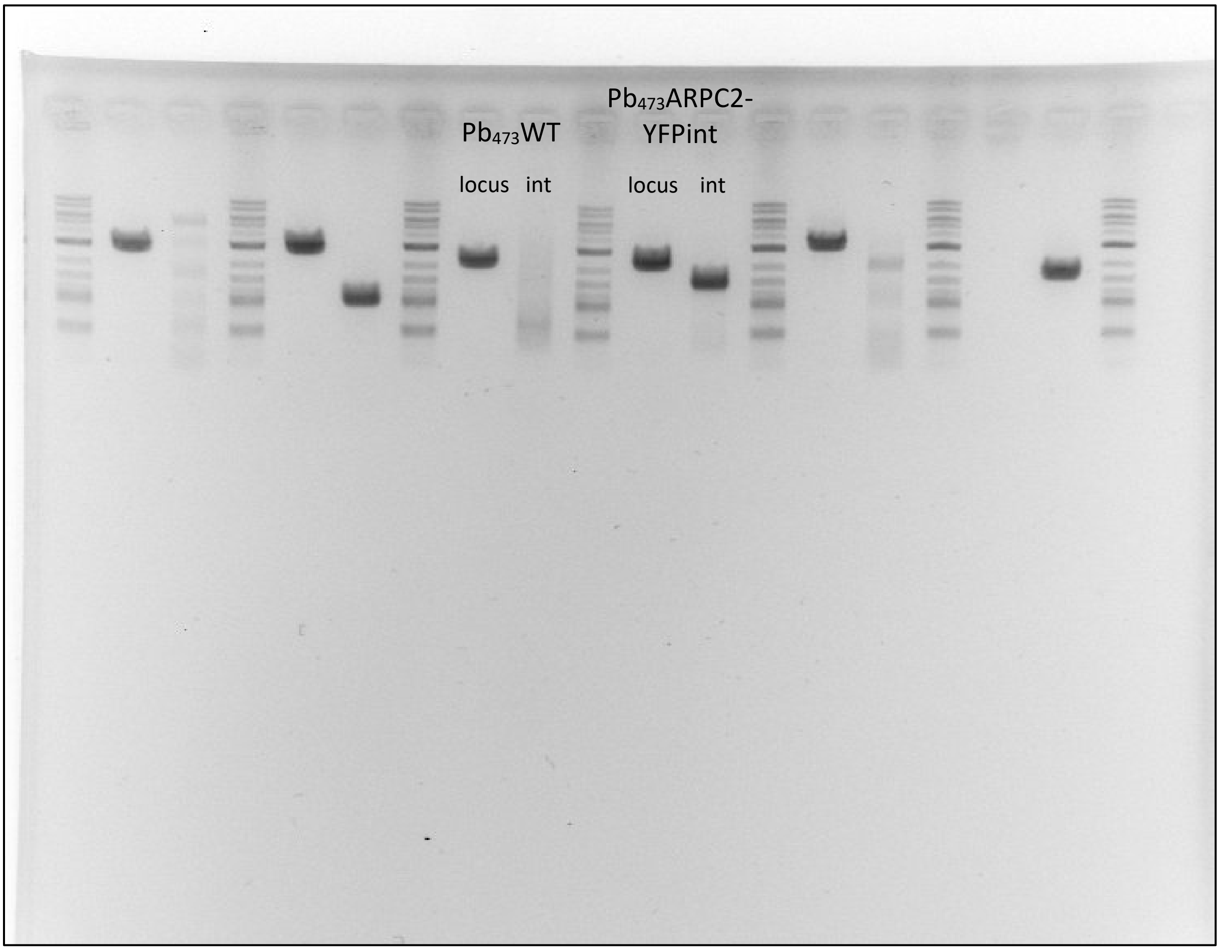

Supplementary Figure 4b - Uncropped gel image. Unlabeled lanes correspond to an unrelated project.

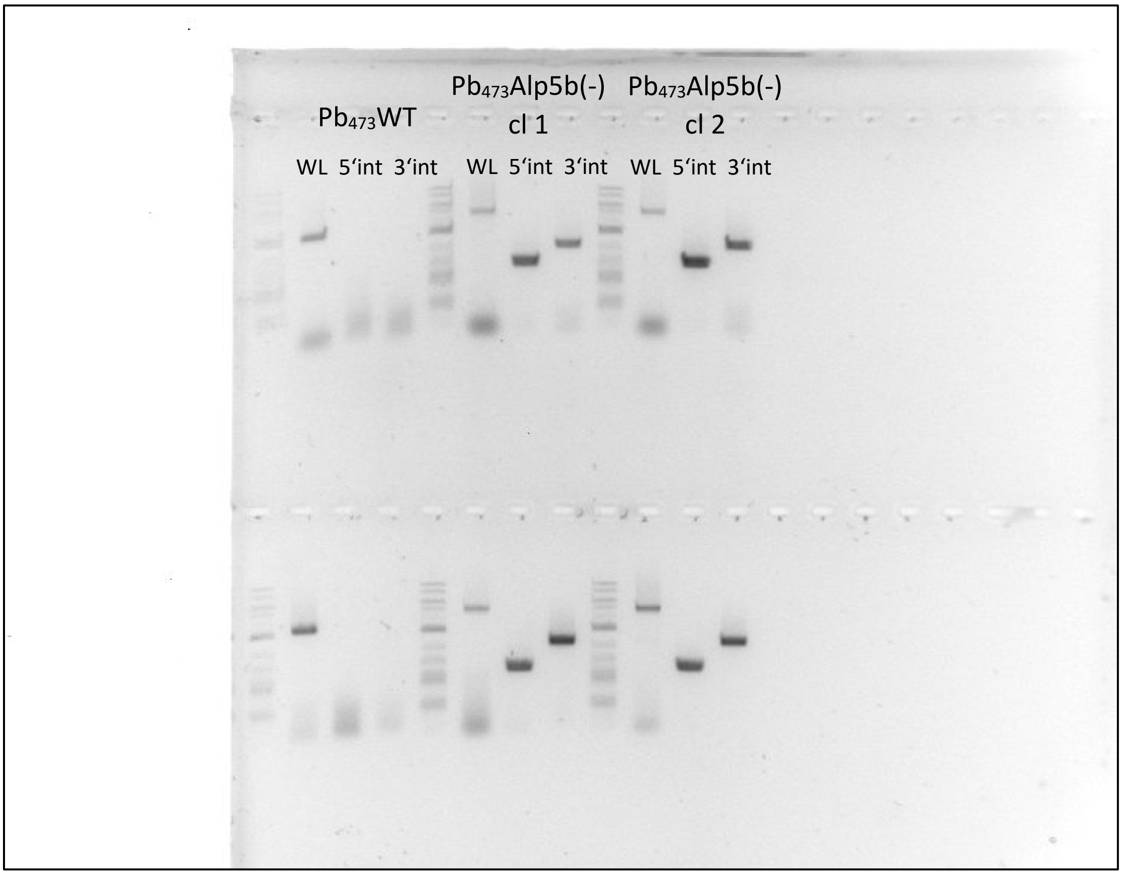

Supplementary Figure 5b - Uncropped gel image. The lanes on the bottom correspond to an unrelated project.

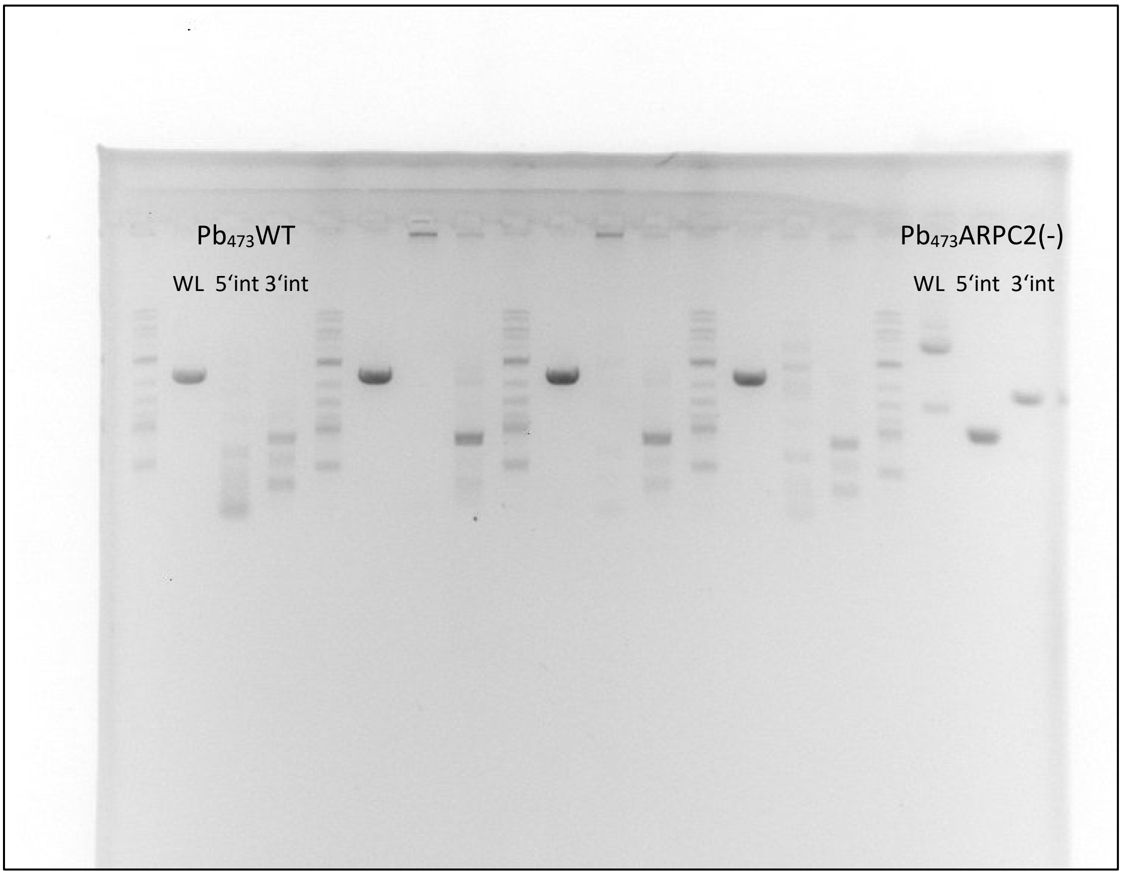

Supplementary Figure 5d - Uncropped gel image. Unlabeled lanes belong to clonal parasite lines that proved to be wildtype and not transgenic parasites.
