# Supplementary material for: An atypical Arp2/3 complex is required for Plasmodium DNA segregation and malaria transmission
Source: Nat Microbiol. 2025 Jun 13;10(7):1775–90. doi: 10.1038/s41564-025-02023-6 (PMC12222016; doi:10.1038/s41564-025-02023-6)
Supplement: Supplementary file 2 — Reporting Summary [file 41564_2025_2023_MOESM2_ESM.pdf]

nature portfolio

Corresponding author(s): Prof.Dr. Matthias Marti
Dr. Franziska Hentzschel

Last updated by author(s): Apr 21, 2025

Reporting Summary

Nature Portfolio wishes to improve the reproducibility of the work that we publish. This form provides structure for consistency and transparency in reporting. For further information on Nature Portfolio policies, see our Editorial Policies and the Editorial Policy Checklist.

Statistics

For all statistical analyses, confirm that the following items are present in the figure legend, table legend, main text, or Methods section.

n/a	Confirmed	
☐	☒	The exact sample size (*n*) for each experimental group/condition, given as a discrete number and unit of measurement
☐	☒	A statement on whether measurements were taken from distinct samples or whether the same sample was measured repeatedly
☐	☒	The statistical test(s) used AND whether they are one- or two-sided<br>*Only common tests should be described solely by name; describe more complex techniques in the Methods section.*
☒	☐	A description of all covariates tested
☐	☒	A description of any assumptions or corrections, such as tests of normality and adjustment for multiple comparisons
☐	☒	A full description of the statistical parameters including central tendency (e.g. means) or other basic estimates (e.g. regression coefficient) AND variation (e.g. standard deviation) or associated estimates of uncertainty (e.g. confidence intervals)
☐	☒	For null hypothesis testing, the test statistic (e.g. *F*, *t*, *r*) with confidence intervals, effect sizes, degrees of freedom and *P* value noted<br>*Give P values as exact values whenever suitable.*
☒	☐	For Bayesian analysis, information on the choice of priors and Markov chain Monte Carlo settings
☒	☐	For hierarchical and complex designs, identification of the appropriate level for tests and full reporting of outcomes
☒	☐	Estimates of effect sizes (e.g. Cohen's *d*, Pearson's *r*), indicating how they were calculated

Our web collection on statistics for biologists contains articles on many of the points above.

Software and code

Policy information about availability of computer code

Data collection	Flow cytometry: BD FACSDiva v8.0.1.1<br>Microscopy: Zeiss ZEN v2.6
Data analysis	Most data was plotted and analysed in GraphPad Prism (v10.0.3), except for mass spectrometry data, which was analysed using Proteome Discoverer 2.5 and Perseus (v2.0.10) and plotted with R (v4.2.2). Flow cytometry data was analysed using FlowJo (v10.7.2). ZEN Blue 3.01 software was used for the post-2D or 3D Airyscan processing with automatically determined default Airyscan settings. All images were further processed in FIJI (v2.14.0). Structures were predicted using Alphafold3 and visualised using UCSF ChimeraX (1.9). EM tomograms were joined and reconstructed using IMOD (v4.11.12).

For manuscripts utilizing custom algorithms or software that are central to the research but not yet described in published literature, software must be made available to editors and reviewers. We strongly encourage code deposition in a community repository (e.g. GitHub). See the Nature Portfolio guidelines for submitting code & software for further information.

Data

Policy information about availability of data

All manuscripts must include a data availability statement. This statement should provide the following information, where applicable:

- Accession codes, unique identifiers, or web links for publicly available datasets
- A description of any restrictions on data availability
- For clinical datasets or third party data, please ensure that the statement adheres to our policy

The Mus musculus and Plasmodium berghei protein databases that were used in this study can be accessed at Uniprot (UP000000589 and UP000074855, respectively). Raw mass spectrometry proteomics data have been deposited to the ProteomeXchange Consortium via the PRIDE partner repository82 with the dataset identifiers PXD046181, PXD051260 and PXD060997. All other data are available included in the main text orarticle and the supplementary information. Source data are provided with this paper. materials. Materials are available upon request.

Research involving human participants, their data, or biological material

Policy information about studies with human participants or human data. See also policy information about sex, gender (identity/presentation), and sexual orientation and race, ethnicity and racism.

Reporting on sex and gender	No research involving human participants, their data, or biological material
Reporting on race, ethnicity, or other socially relevant groupings	No research involving human participants, their data, or biological material
Population characteristics	No research involving human participants, their data, or biological material
Recruitment	No research involving human participants, their data, or biological material
Ethics oversight	No research involving human participants, their data, or biological material

Note that full information on the approval of the study protocol must also be provided in the manuscript.

Field-specific reporting

Please select the one below that is the best fit for your research. If you are not sure, read the appropriate sections before making your selection.

☒ Life sciences ☐ Behavioural & social sciences ☐ Ecological, evolutionary & environmental sciences

For a reference copy of the document with all sections, see nature.com/documents/nr-reporting-summary-flat.pdf

Life sciences study design

All studies must disclose on these points even when the disclosure is negative.

Sample size	No statistical method was used to predetermine sample size. Sample sizes were chosen based on previous experience with similar experiments allowing for robust replication while minimizing usage of rodents. The sample size for mouse experiments was thus 3-5 mice per group. For mosquitoes, 10-30 mosquitoes per timepoint were collected and analysed. For microscopy, 10-25 images per condition/replicate were collected and all cells on each image were quantitatively analysed. Exact sample size is provided in the figure legend of each experiment.
Data exclusions	No data was excluded.
Replication	Most experiments were performed at least in biological triplicates on different days. Selected experiments were performed in biological duplicates, as indicated in the respective figure legends. No unexpected variation between replicates was observed.
Randomization	Mice were age-matched and were allocated randomly to each group. All other samples were allocated in random to experimental groups.
Blinding	Given the nature of the experiments, it was not possible to blind investigators to mouse experimental groups during experimental assays and sample generation, as mouse infection and sample processing had to be handled by the same investigator. To avoid bias during quantitative data analysis as much as possible, microscopy images (with the exception of oocyst number and size determination) were blinded after acquisition and thus analysed in a single-blinded fashion.

Reporting for specific materials, systems and methods

We require information from authors about some types of materials, experimental systems and methods used in many studies. Here, indicate whether each material, system or method listed is relevant to your study. If you are not sure if a list item applies to your research, read the appropriate section before selecting a response.

Materials & experimental systems

n/a	Involved in the study	
☐	☒	Antibodies
☐	☒	Eukaryotic cell lines
☒	☐	Palaeontology and archaeology
☐	☒	Animals and other organisms
☒	☐	Clinical data
☒	☐	Dual use research of concern
☒	☐	Plants

Methods

n/a	Involved in the study	
☒	☐	ChIP-seq
☐	☒	Flow cytometry
☒	☐	MRI-based neuroimaging

Antibodies

Antibodies used

Immunofluorescence: rabbit-anti-GFP (1:50 dilution, Invitrogen, G10362), mouse-anti-tubulin (1:1000 dilution, Sigma-Aldrich, T5168-2ML), rat-anti-HA (1:1000 dilution, Roche, 11867423001), Alexa-546-anti-rabbit (Invitrogen, A11035), Alexa-488-anti-mouse (Invitrogen, A11029), Alexa-488-anti-rabbit (Invitrogen, A11008), Alexa-647-anti-rat (BD Pharma, 51-9006589), mouse-anti-PbHsp70 (clone 2E6 generated by Tsuji et al., PMID 8153120)

Validation

All antibodies used were standard commercial or previously published antibodies and have been validated by the respective manufacturer for specificity. In addition, for all immunofluorescence assays, appropriate controls (untagged wildtype cells or secondary antibody-only controls) were run in parallel to ensure specificity of the signal.

rabbit-anti-GFP (1:50 dilution, Invitrogen, G10362): Validated by manufacturer for use in immunofluorescence assays (https://www.thermofisher.com/antibody/product/GFP-Antibody-Recombinant-Monoclonal/G10362).

mouse-anti-tubulin (1:1000 dilution, Sigma-Aldrich, T5168-2ML): Validated by manufacturer for use in immunofluorescence assays (https://www.sigmaaldrich.com/DE/de/product/sigma/t5168). Used in multiple publications to stain Plasmodium tubulin (e.g. PMID 36154191 or PMID 37704606)

rat-anti-HA (1:1000 dilution, Roche, 11867423001): Validated by manufacturer for use in immunofluorescence assays (https://www.sigmaaldrich.com/DE/de/product/roche/roahaha)

Alexa-546-anti-rabbit (Invitrogen, A11035): Validated by manufacturer for use in immunofluorescence assays (https://www.thermofisher.com/antibody/product/Goat-anti-Rabbit-IgG-H-L-Highly-Cross-Adsorbed-Secondary-Antibody-Polyclonal/A-11035)

Alexa-488-anti-mouse (Invitrogen, A11029): Validated by manufacturer for use in immunofluorescence assays (https://www.thermofisher.com/antibody/product/Goat-anti-Mouse-IgG-H-L-Highly-Cross-Adsorbed-Secondary-Antibody-Polyclonal/A-11029)

Alexa-488-anti-rabbit (Invitrogen, A11008): Validated by manufacturer for use in immunofluorescence assays (https://www.thermofisher.com/antibody/product/Goat-anti-Rabbit-IgG-H-L-Cross-Adsorbed-Secondary-Antibody-Polyclonal/A-11008)

Alexa-647-anti-rat (BD Pharma, 51-9006589): Validated internally by using wildtype cells not expressing the HA - Tag against which the primary antibody was directed. No unspecific signal was observed.

mouse-anti-PbHsp70 (clone 2E6): Validated by Tsuji et al., PMID 8153120, and used in >150 citations.

Eukaryotic cell lines

Policy information about cell lines and Sex and Gender in Research

Cell line source(s)	Human HepG2, source ATCC, Identifier HB-8065
Authentication	None of the cell lines were authenticated
Mycoplasma contamination	Cell lines were not tested for mycoplasma contamination
Commonly misidentified lines (See ICLAC register)	No commonly misidentified lines were used in this study.

Animals and other research organisms

Policy information about studies involving animals; ARRIVE guidelines recommended for reporting animal research, and Sex and Gender in Research

Laboratory animals

Mice (Mus musculus), outbred: Theiler original or CD1, inbred: C57Bl/6 ; female; 5-8 weeks old. Mice were kept in groups of 2 to 4

Laboratory animals | mice per cage under specified pathogen-free (SPF) conditions within the animal facilities at Heidelberg University or University of Glasgow on a 12 hour light/dark cycle at 22 °C (± 2 °C) and 50-60% humidity with ad libitum access to food and water.

Wild animals | The study did not involve wild animals

Reporting on sex | All mice were female, a commonly used model system to study rodent Plasmodium species. The sex of the mice has no impact on the cell biology of the parasite.

Field-collected samples | The study did not involve samples collected from the field

Ethics oversight | All experiments were performed in accordance with GV-SOLAS and FELASA guidelines and have been approved by the German authorities (Regierungspräsidium Karlsruhe) or according to the guidelines defined by the Home Office and UK Animals (Scientific Procedures) Act 1986 and approved by the UK Home Office (project license P6CA91811) and the University of Glasgow animal welfare and ethical review body.

Note that full information on the approval of the study protocol must also be provided in the manuscript.

Plants

Seed stocks | *Report on the source of all seed stocks or other plant material used. If applicable, state the seed stock centre and catalogue number. If plant specimens were collected from the field, describe the collection location, date and sampling procedures.*

Novel plant genotypes | *Describe the methods by which all novel plant genotypes were produced. This includes those generated by transgenic approaches, gene editing, chemical/radiation-based mutagenesis and hybridization. For transgenic lines, describe the transformation method, the number of independent lines analyzed and the generation upon which experiments were performed. For gene-edited lines, describe the editor used, the endogenous sequence targeted for editing, the targeting guide RNA sequence (if applicable) and how the editor was applied.*

Authentication | *Describe any authentication procedures for each seed stock used or novel genotype generated. Describe any experiments used to assess the effect of a mutation and, where applicable, how potential secondary effects (e.g. second site T-DNA insertions, mosiacism, off-target gene editing) were examined.*

Flow Cytometry

Plots

Confirm that:

☒ The axis labels state the marker and fluorochrome used (e.g. CD4-FITC).

☒ The axis scales are clearly visible. Include numbers along axes only for bottom left plot of group (a 'group' is an analysis of identical markers).

☒ All plots are contour plots with outliers or pseudocolor plots.

☒ A numerical value for number of cells or percentage (with statistics) is provided.

Methodology

Sample preparation | For asexual growth rate and gametocyte production: A drop of blood was collected from the tip of the tail of an infected mouse and stained in 1 ml DRAQ5 (1:1000 diluted in PBS) for 10 min at room temperature. Cells were washed once with PBS before analysis by flow cytometry.
For detection of ARPC1-HA: 100 µl blood were collected from infected mice and fixed by adding equal volume of 4% PFA/0.0075% Glutaraldehyde. Cells were stained according to standard immunofluorescence staining procedures with an antibody against HA (Roche, 11867423001) and GFP (Invitrogen, G10362), and with Hoechst at a final concentration of 10 µg/ml.

Instrument | For asexual growth rate and gametocyte production: MACSQuant VYB flow cytometer
For detection of ARPC1-HA: BD FACSCelesta cell analyzer

Software | FACS Diva Software
FlowJo V 10.7.2

Cell population abundance | No sorting was done.

Gating strategy | For all gating strategies, cells were initially gated on the main population in the FSC/SSC plot to exclude small debris and very large cells, followed by gating on single cells as identified by being on the diagonal of the FSC-H/FSC-A plot. For detection of asexual growth rate and gametocyte production, DRAQ5-positive cells (i.e. all parasites) were further gated on mCherry and GFP (i.e. female and male gametocytes respectively). For quantification of ARPC1-HA expression, parasites were identified by Hoechst-Staining and then gated on mCherry and GFP (i.e. female and male gametocytes, respectively). HA signal was quantified based on median Alexa647 fluorescence intensity in those populations.

☒ Tick this box to confirm that a figure exemplifying the gating strategy is provided in the Supplementary Information.
