# Supplementary material for: An atypical Arp2/3 complex is required for Plasmodium DNA segregation and malaria transmission
Source: Nat Microbiol. 2025 Jun 13;10(7):1775–90. doi: 10.1038/s41564-025-02023-6 (PMC12222016; doi:10.1038/s41564-025-02023-6)

Extended Data Fig 2b – Uncropped gel images

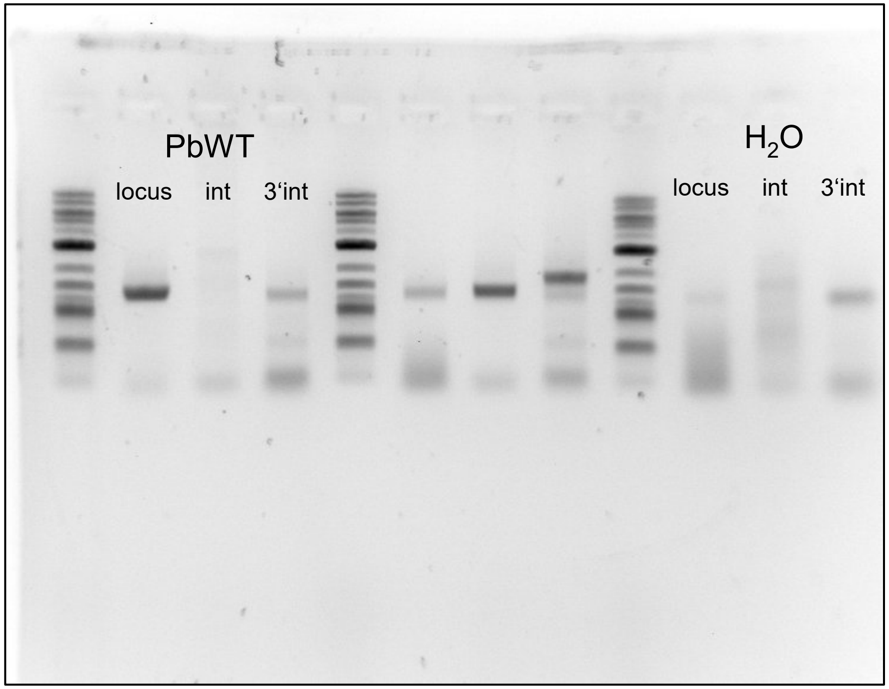

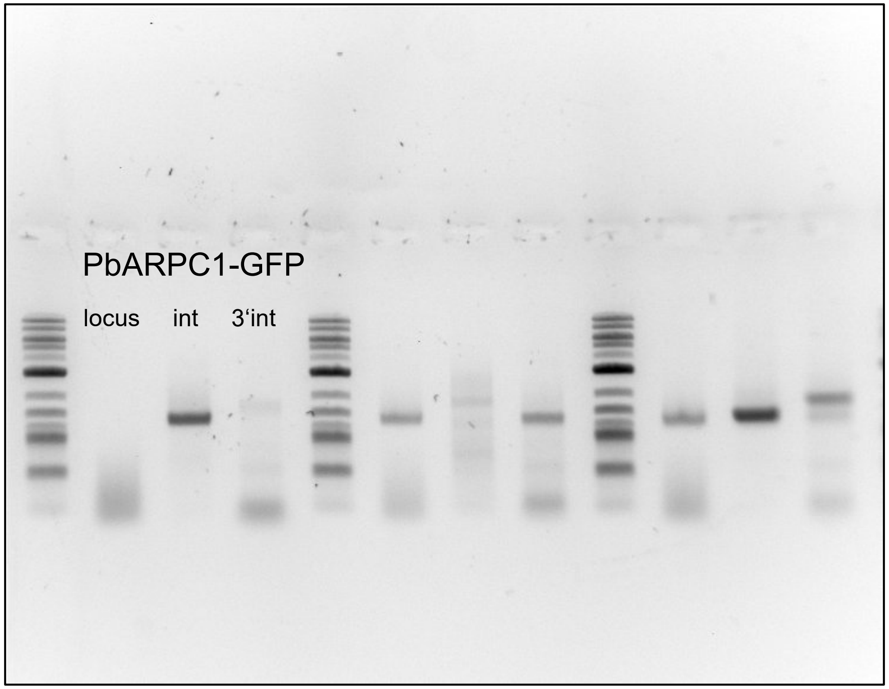

Unlabeled gel lanes are gDNA of different populations of PbARPC1-GFP that were not clonal and thus were not used in study. The third lane in each set belonged to a PCR for integration at the 3’end, which was due to a contamination visible in the water control not interpreted.

Extended Data Fig 3c,d – Uncropped gel image

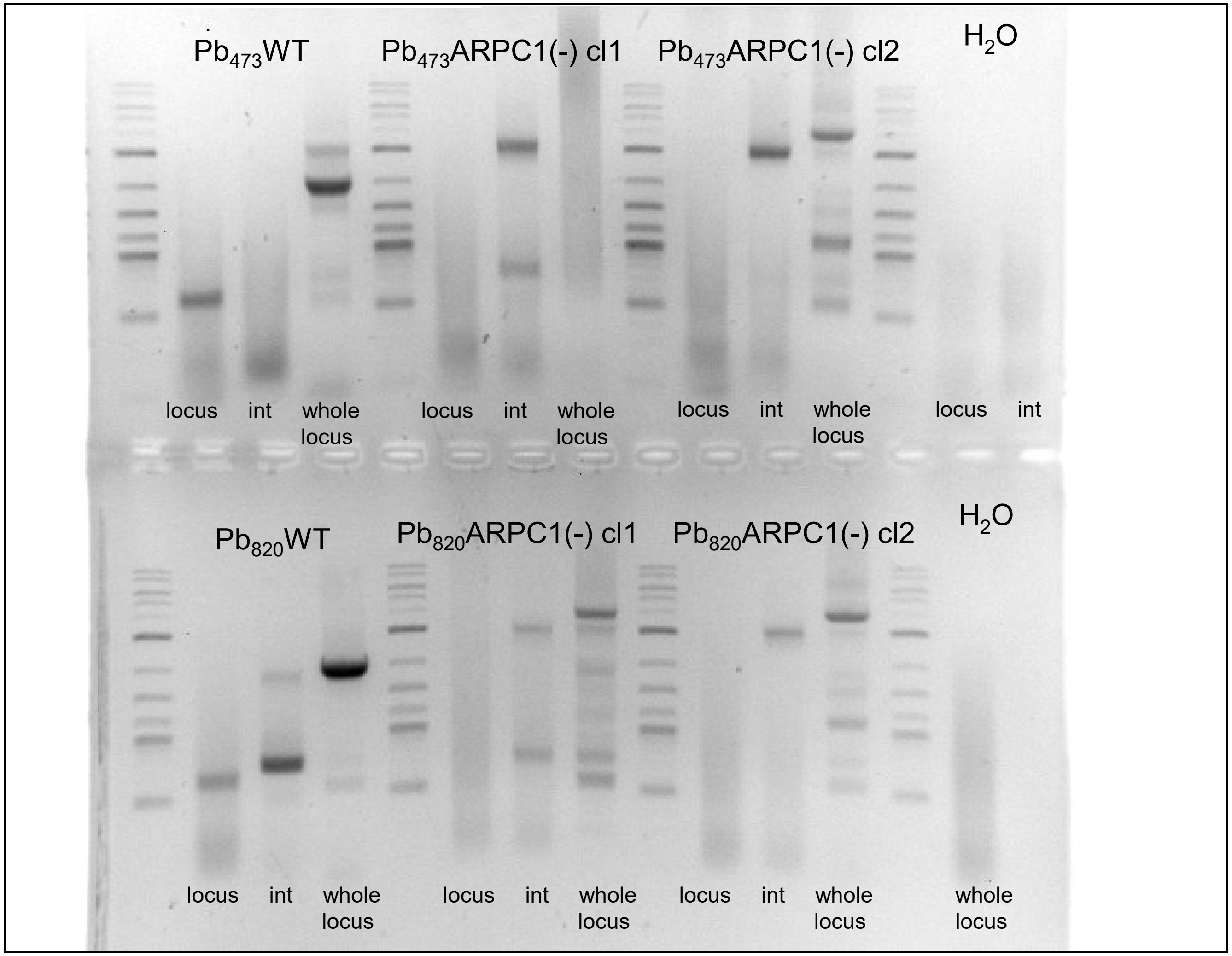

The third lane in each set belonged to a whole locus PCR which was due to the high amount of unspecific amplification not further interpreted.

Extended Data Fig 4b – Uncropped gel image

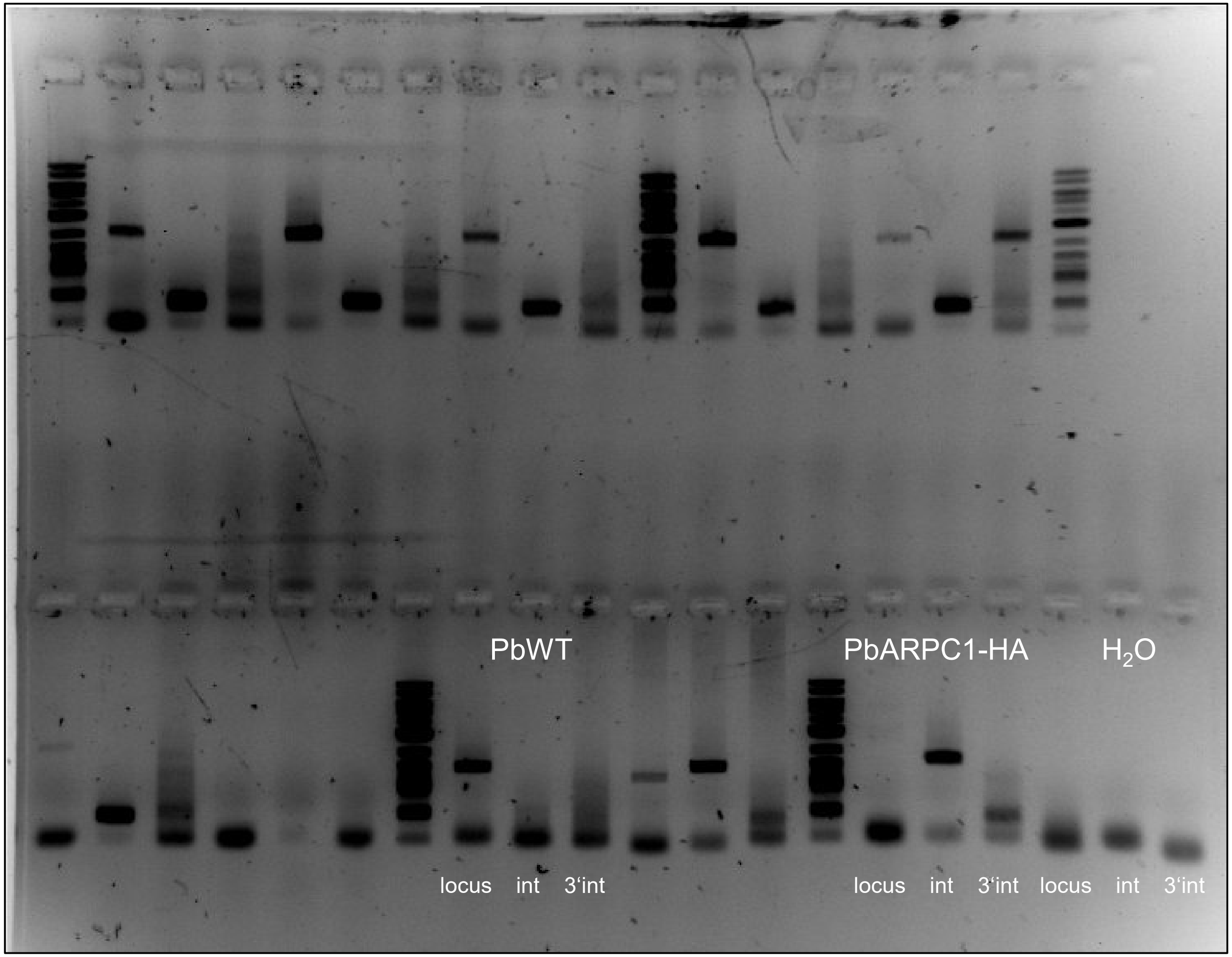

Unlabeled lanes correspond to PCRs from an unrelated project or to a non-clonal population of PbARPC1-HA.

Extended Data Fig 8b – Uncropped gel image

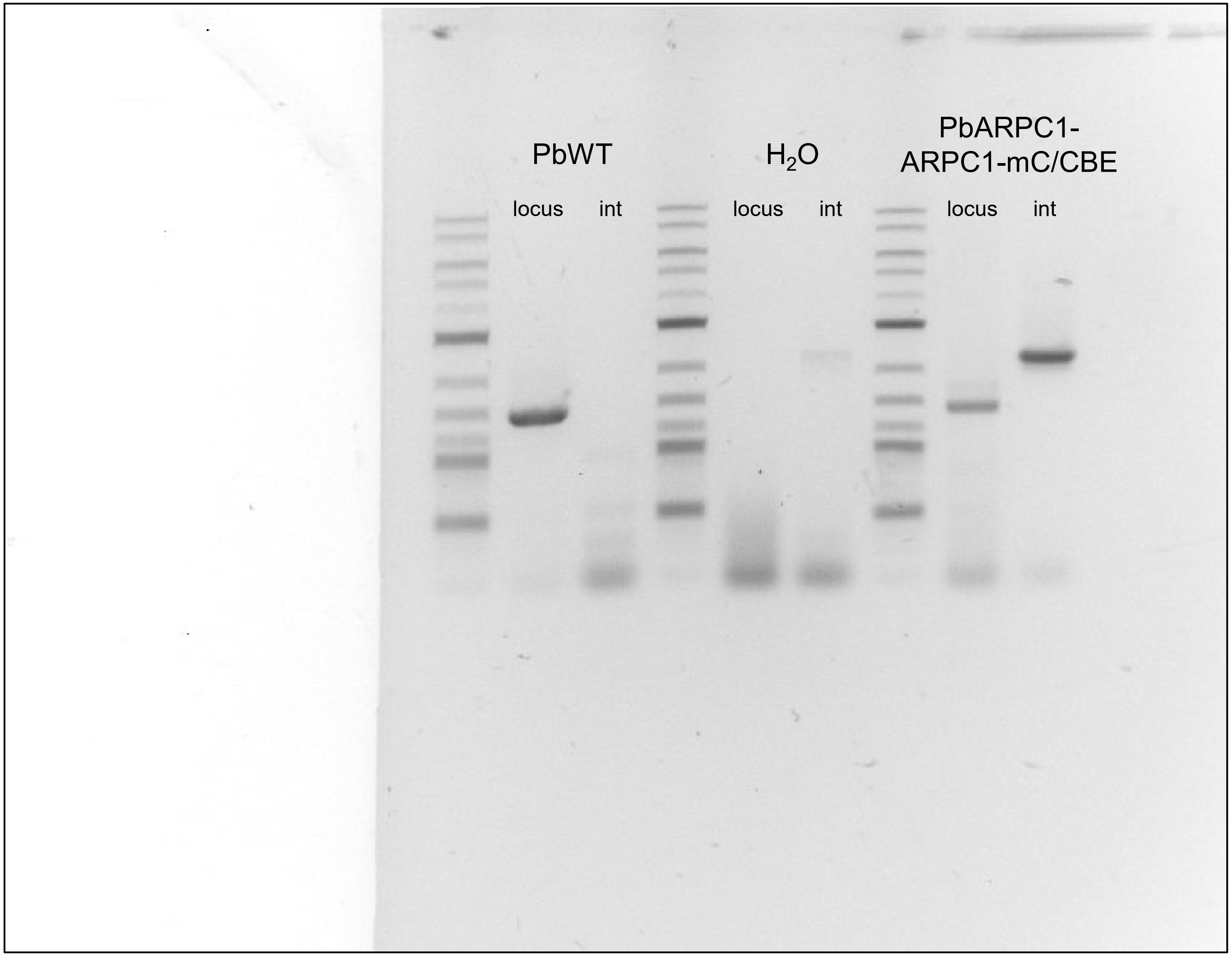

Extended Data Fig 9b – Uncropped gel image

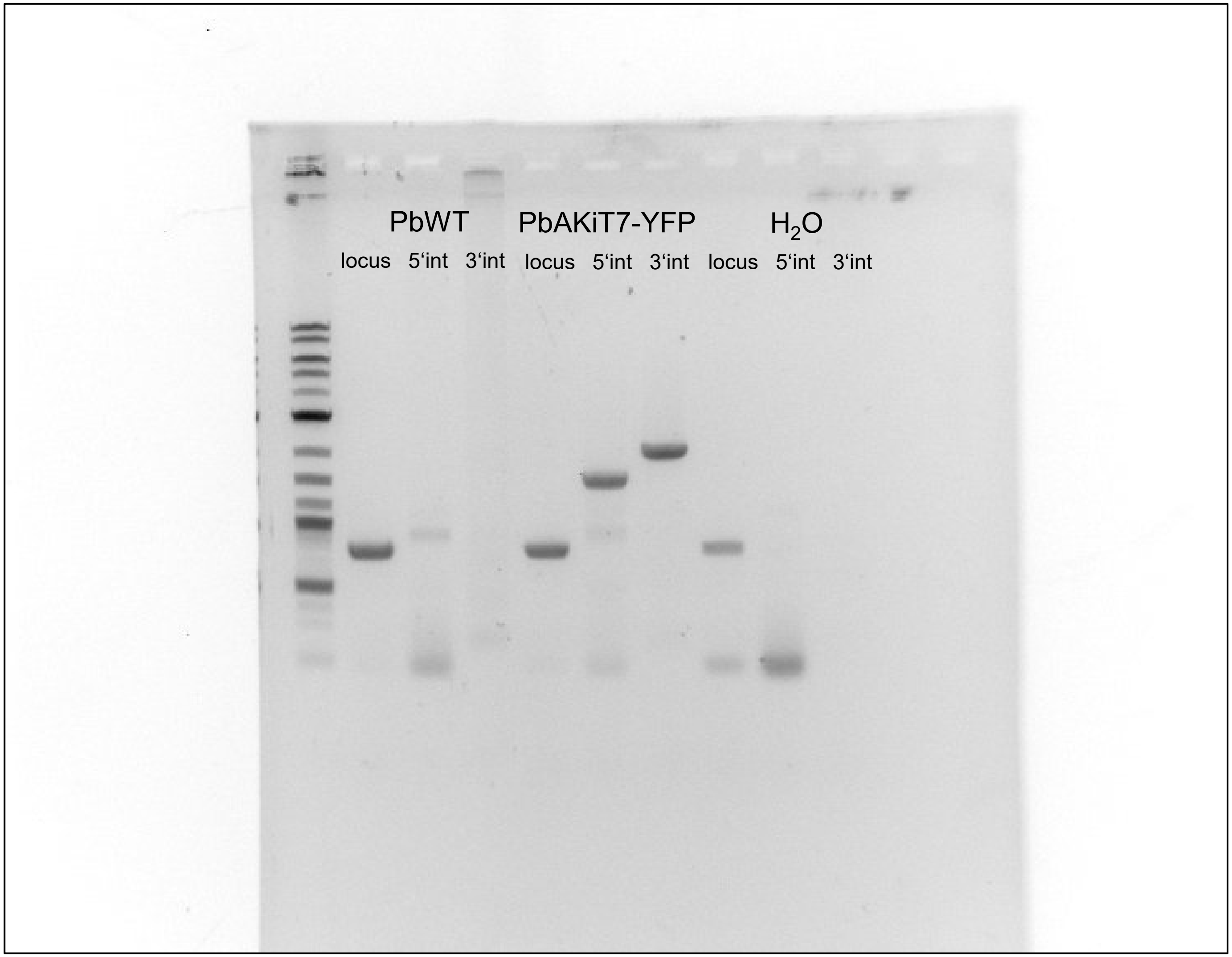

Extended Data Fig 10b – Uncropped gel image

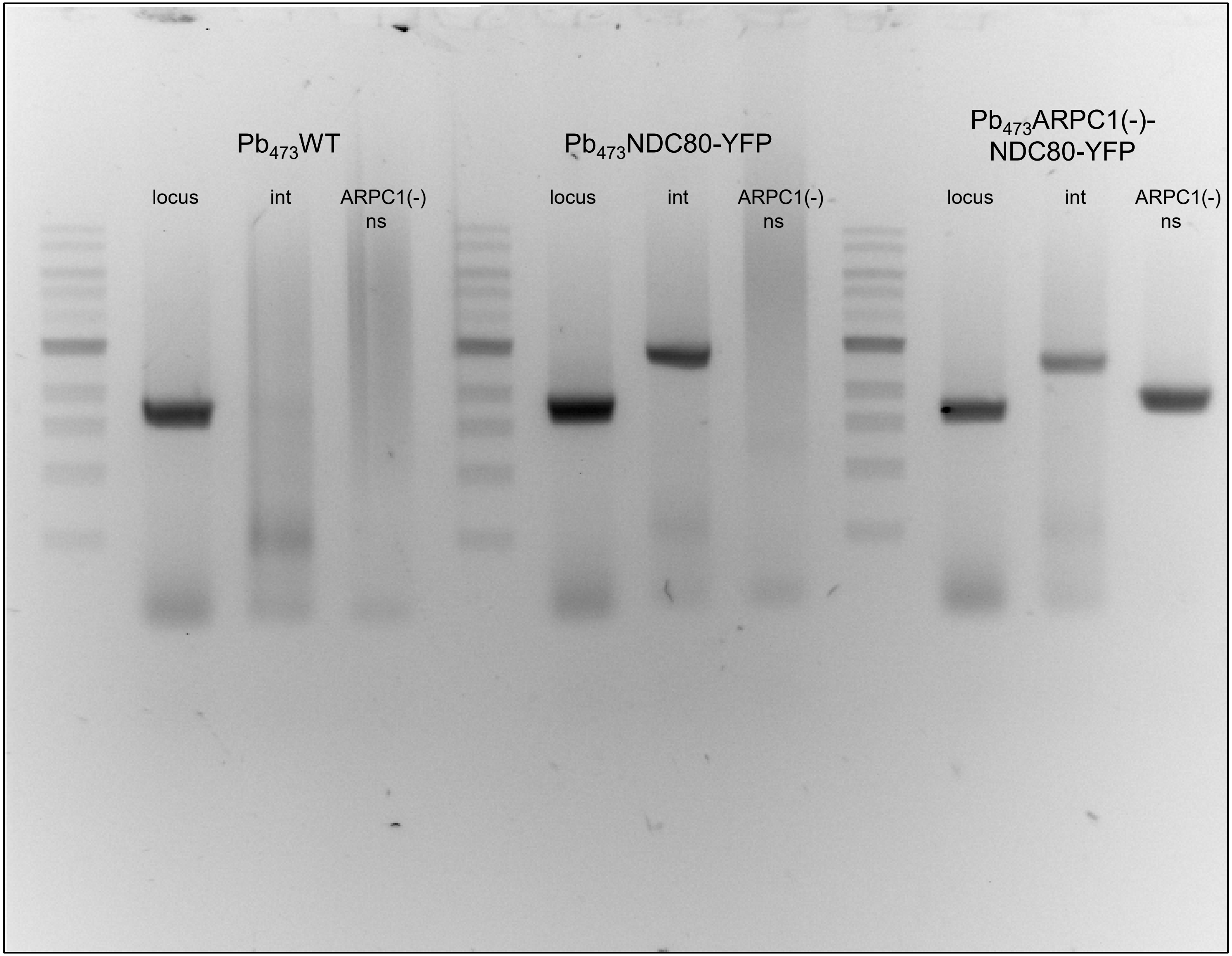

Supplement: Supplementary file 11 — Unprocessed gel images. [file 41564_2025_2023_MOESM11_ESM.pdf]
